# A Comparison of Germany and the United Kingdom Indicates That More SARS-CoV-2 Circulation and Less Restrictions in the Warm Season Might Reduce Overall COVID-19 Burden

**DOI:** 10.3390/life12070953

**Published:** 2022-06-24

**Authors:** David Meintrup, Martina Nowak-Machen, Stefan Borgmann

**Affiliations:** 1 Faculty of Engineering and Management, University of Applied Sciences Ingolstadt, 85049 Ingolstadt, Germany; 2 Department of Anaesthesia and Intensive Care Medicine, Ingolstadt Hospital, 85049 Ingolstadt, Germany; martina.nowak-machen@klinikum-ingolstadt.de; 3 Department of Anesthesiology and Intensive Care Medicine, Teaching Faculty, University Hospital Tuebingen, Eberhard-Karls-University, 72076 Tuebingen, Germany; 4 Department of Infectious Diseases and Infection Control, Ingolstadt Hospital, 85049 Ingolstadt, Germany; stefan.borgmann@klinikum-ingolstadt.de

**Keywords:** COVID-19, SARS-CoV-2, non-pharmaceutical interventions, seasonality, Germany, UK, excess mortality

## Abstract

(1) Background: Between March 2020 and January 2022 severe acute respiratory syndrome coronavirus type 2 (SARS-CoV-2) caused five infection waves in Europe. The first and the second wave was caused by wildtype SARS-CoV-2, while the following waves were caused by the variants of concern Alpha, Delta, and Omicron respectively. (2) Methods: In the present analysis, the first four waves were compared in Germany and the UK, in order to examine the COVID-19 epidemiology and its modulation by non-pharmaceutical interventions (NPI). (3) Results: The number of COVID-19 patients on intensive care units and the case fatality rate were used to estimate disease burden, the excess mortality to assess the net effect of NPI and other measures on the population. The UK was more severely affected by the first and the third wave while Germany was more affected by the second wave. The UK had a higher excess mortality during the first wave, afterwards the excess mortality in both countries was nearly identical. While most NPI were lifted in the UK in July 2021, the measures were kept and even aggravated in Germany. Nevertheless, in autumn 2021 Germany was much more affected, nearly resulting in a balanced sum of infections and deaths compared to the UK. Within the whole observation period, in Germany the number of COVID-19 patients on ICUs was up to four times higher than in the UK. Our results show that NPI have a limited effect on COVID-19 burden, seasonality plays a crucial role, and a higher virus circulation in a pre-wave situation could be beneficial. (4) Conclusions: Although Germany put much more effort and resources to fight the pandemic, the net balance of both countries was nearly identical, questioning the benefit of excessive ICU treatments and of the implementation of NPI, especially during the warm season.

## 1. Introduction

The coronavirus disease 2019 (COVID-19) was recognized as a pandemic by the World Health Organization (WHO) in March 2020 [1]. Two years later, at the time of writing in March 2022, the WHO counted more than 400 million infections and 6 million COVID-19 related deaths. Roughly one third of the infections and deaths occurred in Europe. In most European countries, five waves of infections occurred over the past two years. The first two, beginning in March 2020 and towards the end of the year 2020, were caused by the wildtype of the virus. The variant of concern (VOC) B.1.1.7 (Alpha) started a new wave in England at the beginning of 2021, and then spread all over Europe. The second half of the year 2021 was dominated by the VOC B.1.617.2 (Delta), first detected in India in late 2020. Finally, in November 2021 variant B.1.1.529 (Omicron) spread from South Africa causing the majority of SARS-CoV-2 infections in Europe.

The pandemic forced governments to take measures in order to control the damage caused by COVID-19. These non-pharmaceutical interventions (NPI) reached from simple behavioural recommendations like social distancing and frequent hand washing to drastic lock-downs including curfews. There were two obvious goals that these restrictions should ensure: on the one hand, to keep the number of infections and subsequent deaths caused by COVID-19 reasonably low and, on the other hand, to secure the functioning of the health systems. Despite these common goals, the number, intensity, and length of interventions implemented by governments in Europe varied widely [2].

Another aspect, however, is maybe less obvious but at least equally important on the long run: to reach immunity of a large part of the population. Before vaccines became available, the only way to acquire a certain amount of immunity was through an infection. The currently available vaccines do not provide sterile immunity, hence infections continue to occur in vaccinated and unvaccinated people. If immunity is build by vaccinations and (potentially additional) infections, it is a legitimate question to ask if measures taken to control the spread of the virus can be too restrictive, too strongly preventing contact with the virus and therefore delaying the path to immunity of a population. It was already shown in another analysis, that European countries, which had suffered from high COVID-19 burden in the first wave, were less affected during the second wave and vice versa [3]. Moreover, additional infections do not necessarily couple with more deaths if the infection rate stays below a certain threshold [4]. Both results support the hypothesis that a certain circulation of the coronavirus in a population can be beneficial for the long-term outcome of the pandemic.

We address these questions by comparing Germany, a European country with relatively strict regulations, with the UK, where the implemented measures were less severe. In the public and published opinion, Germany was considered a role model from the beginning [5], while the approach of the UK was strongly criticized [6]. We believe that a more detailed and careful data analysis is needed and might lead to different conclusions.

In the present work, we sought to compare the course of the pandemic in the UK and Germany at different levels. Latest in the current omicron (VOC B.1.1.529) wave it became obvious that each mutation has its own pathogenicity [7,8]. Hence, each fair comparison has to be stratified by the mutant type. We will consider the four waves (two caused by the wildtype, the alpha, and the delta wave) that occurred in both countries during the first two years of the pandemic, taking into account seasonality as an important factor. We will look at laboratory confirmed infections, COVID-19 related deaths, and case fatality rates per period and compare them between the two countries. As these measures might be affected by confounding variables as test rates and similar effects, we will also compare the excess mortality. It is an important metric because it includes both undetected COVID-19 deaths and other causes of death, indirectly caused by the pandemic.

The German health system is known to be one of the most expensive in the world. The hospital beds and ICU capacities outrun the numbers of most other European countries, including the United Kingdom. In a health crisis like the current pandemic, this might lead to higher costs, but should certainly provide a considerable advantage over other countries in the fight against the coronavirus. Therefore, we will also look at the question if the higher number of ICU beds and patients in Germany compared to the UK proved to be beneficial for the German population.

## 2. Methods

Our work is based on the publicly available data set provided by the authors of the website “Our World in Data” [9]. They use several publicly available sources to collect their data, including the COVID-19 Data Repository by the Center for Systems Science and Engineering (CSSE) at Johns Hopkins University [10]. The complete Our World in Data COVID-19 data set can be downloaded from their GitHub repository [11]. We used the following time series for Germany and the UK: incidences, deaths, ICU patients, excess mortality P-score, cumulative excess mortality. Smoothing for COVID-19 incidences and related deaths was done using a 7-day-moving average.

The excess mortality data tracker of “Our World in Data” uses the numbers of the World Mortality Dataset created by Karlinsky and Kobak [12]. These researchers developed a regularly updated open access all-cause module providing data from all over the world. This module imports the dataset from EuroStat [13] and the Short Term Mortality Database [14]. Both data sets disaggregate by sex and age groups.

Overall, we examined the period from 1 March 2020, the beginning of the pandemic, to the end of 2021. During this time, there were four incidence waves in Germany and in the UK respectively. The first two were caused by the wild type of the virus (Wild 1 and Wild 2), the third by the Alpha variant (Alpha period), and the fourth by the Delta variant (Delta period).

Our approach was not to compare Germany and England over fixed periods of time, but rather with respect to these four periods. We set the end of the first period to 1 August 2020. At that time, the first wave was over in both countries, the second wave had not yet started, and infection rates were low. We defined the end for all other periods when a new variant had become dominant. We considered a new variant as dominant, once more than 50% of the sequencing was assigned to the new variant. For Germany, this information is published in reports by the Robert-Koch-Institut (RKI) [15], for England in the technical briefings of the UK Health Security Agency [16].

Once the four periods were fixed, we aggregated the data for deaths, incidences, ICU patients, and excess mortality for each country and period. Then we calculated the relative statistics Case Fatality Rate (CFR), deaths per million inhabitants, ICU patient days per 1000 incidences, and deaths per 100 ICU patient days, each per country and period.

Time elapses between infection, admission to the intensive care unit if necessary, and potentially death, depending on the reporting system and the course of the disease. Therefore, we estimated shift factors for Germany and the UK, by fitting the time series of incidences to the ICU patients and COVID-19 related deaths, using a minimum variance approach. We took these estimated shift factors into account, when we aggregated the COVID-19 related deaths and ICU patients for each of the four periods.

Over the course of the pandemic, governments implemented restrictive measures in order to control the spread of the coronavirus. In order to compare Germany and the UK, these restrictions have to be taken into account. For Germany, we used the timeline of measures published by the German Federal Ministry of Health [17]. The same information for the UK is gathered by the Institute for Government [18].

## 3. Results

### 3.1. Demography and Public Health

With 83 million people, Germany is about one fifth larger than the UK (68 million people). The age distributions in both countries are similar [19,20]. Germany’s population is a bit more shifted towards elderly people (median age 46.6 versus 40.8 in the UK). The mean life expectancy of 81.3 years is identical (see Table 1). Both health systems belong to the most expensive ones in the world [21,22]. The €411 billion spent in Germany and the €320 billion (269 billion Pound) in the UK in 2020 correspond to roughly 13% of the Gross Domestic Product (GDP) respectively. Per person, this corresponds to €5298 in Germany and €4735 (£3944) in the UK respectively.

However, the supply of hospital beds varies substantially. While Germany has 5.8 hospital beds per 1000 inhabitants, the UK has only about one third-2.1 beds per 1000 inhabitants. The difference is even bigger for intensive care beds. Germany has up to 25,000 ICU beds (close to 30 per 100,000 inhabitants), while the UK has only 4500 (6.6 per 100,000 inhabitants). These figures put Germany at the top of the European ranking in terms of the availability of intensive care [23].

### 3.2. COVID-19 Incidences, Deaths, and Case Fatality Rates

Each mutation of the coronavirus has its own characteristics, therefore separate statistics are provided for the wildtype, the Alpha, and the Delta variant. As the wildtype caused two waves, we further split the data for the wildtype into two periods (denoted “Wild 1”, “Wild 2”), and choose 1 August 2020, as the beginning day for the second period. Table 2 contains the number of COVID-19 cases and deaths, the case fatality rates (CFR), and the number of deaths per million for the five considered periods and the two countries under investigation. In addition, Table 2 contains the total number of days that ICU beds were occupied with COVID-19 patients (“ICU sum”), the number of ICU bed days per 1000 confirmed COVID-19 patients (“ICU per 1000 cases”), and the number of COVID-19 related deaths per 100 ICU bed days (“Deaths per 100 ICU days”).

**Figure 1:**
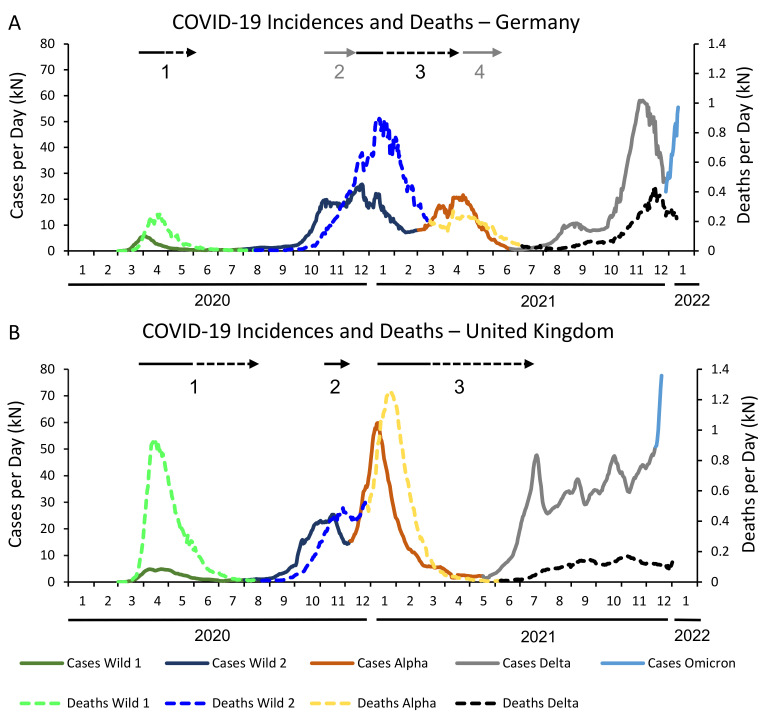
Number of individuals with new identified SARS-CoV-2 infection (solid lines) and deceased COVID-19 individuals (dotted lines) per day in Germany (**A**) and the United Kingdom (**B**) (smoothed numbers). Arrows: Lockdown periods. Dotted lines: Periods in which lockdown measures were only gradually and/or regionally lifted. Grey lines: “Lockdown light” (2) and the “federal emergency brake” (4) in Germany.

Figure 1 displays the COVID-19 incidences and related deaths over the first two years of the pandemic. The colors correspond to the waves and mutations respectively. In Germany, the first wave reached a maximum of almost 6000 cases per day by beginning of April, only one week after the implementation of the first lockdown. The CFR of 4.39% in this first period was relatively low, compared with the rest of Europe. In the UK, however, despite also applying a lockdown end of March, the number of cases only started to drop beginning of May 2020. At that time, the UK had three times more cases per day than Germany, and it’s CFR of 13.62%–over 40,000 COVID-19 related deaths during the first wave compared to 9200 in Germany–was amongst the highest in Europe.

The course of the second wave was very different in both countries. In Germany, it lasted until the first of March 2021, which means that the winter was included. This led to over 2 million confirmed cases and more than 60,000 deaths, resulting in a CFR of 2.87%. In the UK, the second wave ended 6 December 2020, as the alpha variant became dominant. With 1.4 million cases and a CFR of 2.06% it was rather mild, both compared to the first wave in the UK and the second wave in Germany. Interestingly, if one considers the complete period of the wildtype of the coronavirus in both countries, the overall numbers are not that different anymore, resulting in a CFR of 3% in Germany and 4.1% in the UK, despite the huge number of deaths in the UK at the very beginning of the pandemic.

In the UK the first five isolates of the Alpha variant (B.1.1.7) were detected by 15 November 2020. On 7 December 2020 the percentage of this variant of all isolates was over 50%, resulting in a circulation period of 22 days before becoming the dominant variant [16]. The Alpha variant caused a third wave of high infection rates culminating in mid of January 2021. With approximately 2.7 million incidences and 57,000 deaths it reached a CFR of 2.09%. In Germany, the winter 20/21 was still dominated by the wild type. The alpha wave started beginning of March 2021, 1.27 million infections and 17,000 deaths were attributed to it, resulting in a CFR of 1.36%. In Germany, the circulation period had been 57 days [15], twice as long than in the UK (4 January 2021–2 March 2021).

The Delta variant (B.1.617.2) originated in India and became the dominant variant in the UK by mid of May 2021. Despite 6.3 million laboratory confirmed infections, it caused only 21,000 deaths (CFR of 0.33%) by the end of the year 2021, as vaccinations became widely available. By end of August 2021, 70% of the British population got at least one vaccination, compared to 65% in Germany. In Germany, Delta became the dominant variant one month later than in the UK, on 22 June 2021. With only half the amount of infections (3.2 million), but still more than 23,000 COVID-19 related deaths by the end of the year 2021, the CFR = 0.7% of the Delta period was twice as high in Germany as in the UK.

### 3.3. Government Responses to COVID-19

In the UK the first NPI started on 12 March 2020, when cased based isolation became mandatory. Social distancing was encouraged on 16 March 2020, and public events were banned on 26 March 2020 [24]. The first lockdown started on 26 March 2020, and was gradually lifted from 1 June to 14 August. The second lockdown was into force from 5 November 2020, to 2 December. Between those national lockdowns there were local lockdowns in Leicester and parts of Leicestershire. Furthermore, a three-tier system was implemented with various rules depending on local virus burden [25]. The third lockdown began on 6 January 2021. Measures were successively lifted beginning on 8 March 2021, when schools reopened and two individuals were allowed to meet outdoors.

For further opening, a four-step roadmap was created containing reliefs coming into effect in dependence of the epidemiological situation [26]. Initially, step four had been scheduled to be proclaimed on 21 June 2021 [27]. However, to respond to the rapidly spreading Delta variant the deadline was delayed by four weeks to increase the vaccination rate. Eventually, most restrictions of social contacts were lifted on 19 July 2021. For autumn and winter the government announced a two-step program called “Plan A” and “Plan B”. Plan A comprised five parts aiming to prevent the National Health System (NHS) to come under pressure: pharmaceutical interventions (vaccines, drugs), limiting transmission (test, trace and isolate), NHS and social care support, advising people on protection measures, pursuing an international approach (world vaccination, minimizing risks at borders). Plan B described further measures reducing transmission numbers while keeping the economic and social impact low [28]. On 10 December 2021, masks became mandatory for most public indoor activities and on 15 December 2021, the NHS COVID Pass was compulsory in nightclubs and in similar settings. These last measures were directed towards reducing the spread of the Omicron variant (B.1.1.529), which was dominant in the UK by mid of December 2021.

In Germany, government prevention measures were largely comparable to those in the UK until the summer of 2021. On 6 March 2020, case based self isolation became mandatory, social distancing was encouraged on 12 March 2020, and public events were banned on 22 March 2020 [24]. The first nationwide lockdown was imposed from 27 March 2020, to 2 June. After the rise of infection numbers a so-called “lockdown light” was proclaimed on 2 November 2020. Due to further rising infection numbers, Germany went into a second national lockdown on 16 December 2020. Some measures were partially lifted on 10 January 2020, while other restrictions continued for many weeks depending on incidences and the federal state to which a locality belonged. After decreasing incidences in February 2021, they increased again in March and April 2021 due to the arrival of the Alpha variant. As a response to this third wave, another “lockdown light” called the “Federal Emergency Brake” was put into effect, accompanied by stricter limits on social contacts and a curfew from 22:00 pm to 5:00 am. The last day that restrictions based on the “Federal Emergency Brake” were enforced in a German district was 11 June 2021 [17,29].

In the UK, most limitations had been cancelled in summer 2021. In contrast, far-reaching restrictions were introduced in Germany on 23 August 2021, and intensified on 24 November 2021. Among other measures, practically all publicly accessible rooms could only be entered with a vaccination, a negative test result, or after recovery from a COVID-19 infection (so-called “3G-rule”). In addition, visitors of publicly accessible areas had to wear a particle filtering half mask (“FFP2 mask”). Employees were required to work from home. If home office was not possible, fully vaccinated people had to get tested at work twice a week, unvaccinated persons daily. These restrictions were in force at least until 19 March 2022. In contrast to these wide-ranging restrictions, all UK preventive measures ceased on 24 February 2022, and the end of mass testing was announced for 1 April 2022 [30].

As shown in Figure 1, in both countries a full lock-down scenario came into effect in March 2020. The subsequent drop in SARS-CoV-2 infections was seen by many policymakers as evidence of the effectiveness of the interventions. However, imposing only limited restrictions seemed less effective. For example, in Germany the Alpha variant spread in March and April 2021 despite the fact that parts of the measures from the second lock-down were still in force, and in the UK the Delta variant expanded in June and July 2021 although measures of the third lock-down were partially valid.

### 3.4. COVID-19 Related Patients in ICUs and Related Deaths

**Figure 2:**
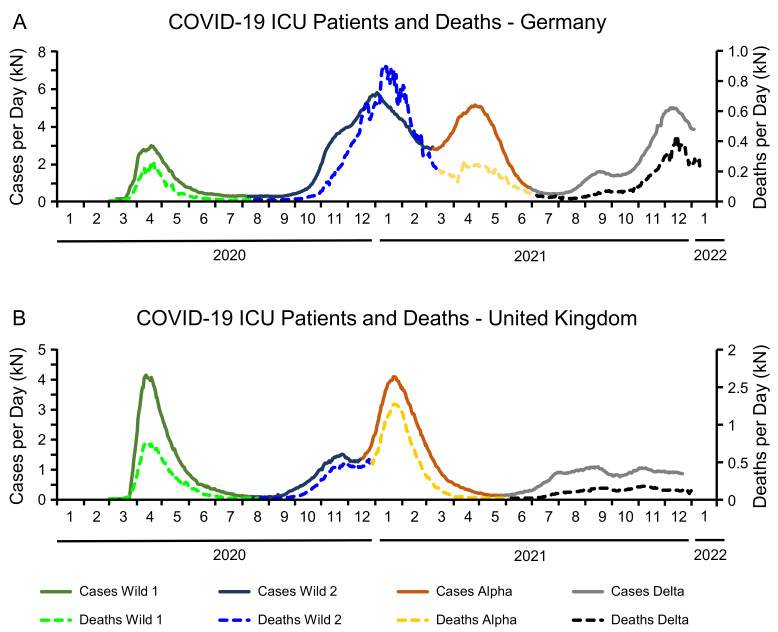
Number of COVID-19 patients treated on an intensive care unit (ICU-solid lines) and number of deceased COVID-19 patients per day in Germany (**A**) and the United Kingdom (**B**).

The time series of COVID-19 ICU patients per day and COVID-19 related deaths for Germany and the UK are depicted in Figure 2. Observing the course of these curves the following conclusions can be drawn:

- Generally, the graph of the ICU patients and COVID-19 related deaths are highly correlated. Modeling the death curve as percentage of the ICU patients plus a constant shift for each wave individually yielded $R^{2}$-values between 90% and 99%.
- Germany tends to have more patients on ICUs. This can be confirmed by the column “ICU sum” in Table 2.
- In both countries, the number of deaths per day reaches its absolute maximum in January 2021, independently of the variant. This peak is caused by the second wave of the wildtype in Germany, and by the Alpha variant in the UK.
- During the Delta period, ICU patients and deaths remained relatively constant in the UK, while both curves exhibited a steep increase in Germany.

**Figure 3:**
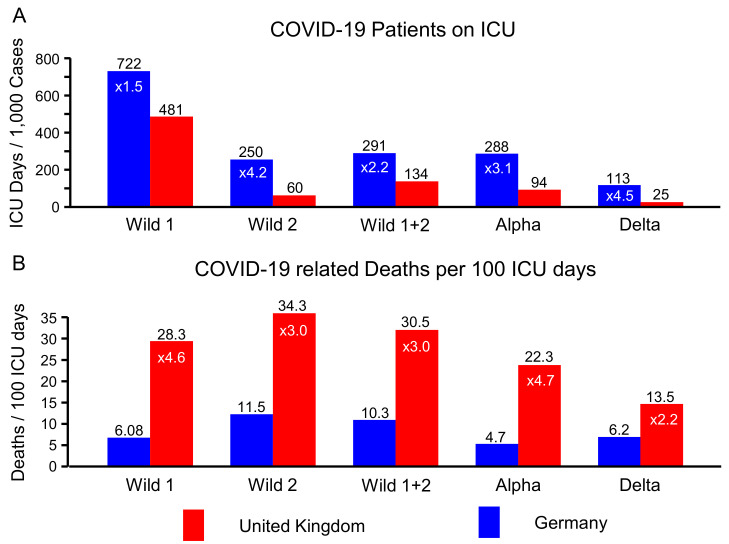
Number of intensive care unit (ICU) days per 1000 COVID-19 cases (**A**) and number of deceased COVID-19 patients per 100 ICU days of COVID-19 patients (**B**) per COVID-19 wave (Wild 1, Wild 2, Wild 1 + 2, Alpha variant, Delta variant) in Germany and the United Kingdom. Numbers (white) inside of bars indicate the factors between adjacent bars.

The number of ICU patient days per 1000 cases with a laboratory confirmed infection (“ICU per 1000 cases” in Table 2) is displayed in Figure 3A. In all periods, Germany has much higher ICU days per 1000 cases. The ratio spans from roughly two-fold to nearly five-fold and is also indicated in Figure 3A. For instance, during the Delta period the UK had 24.5 ICU days per 1000 cases, while Germany had 113, which is 4.6 times more. As a side note, this corresponds quite exactly to the ratio of availabilities of ICU beds in both countries (see Table 1).

The COVID-19 related deaths per 100 ICU patient days (“Deaths per 100 ICU days” in Table 2) is shown in Figure 3B. In order to facilitate the comparison, we turned the order of the countries around when we took the ratios for the deaths per 100 ICU days. Consequently, the ratios again spread between two-fold and nearly five-fold, now with the UK having the higher numbers (see Figure 3B). For example, in the Delta period Germany had 6.2 deaths per 100 ICU days, while the UK had 13.5, which is 2.2 times more.

### 3.5. Excess Mortality

**Figure 4:**
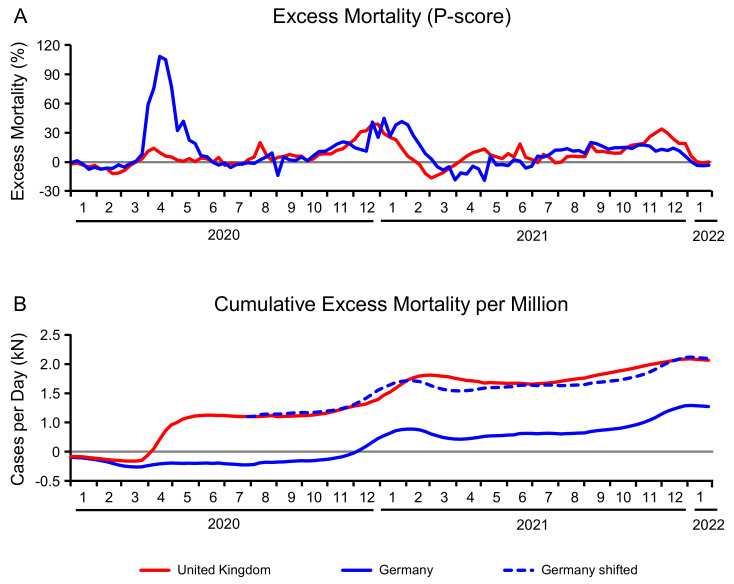
Excess mortality per day in Germany (solid blue line) and the United Kingdom (solid red line). Percentage deviation from the average mortality (P-score) starting 1 January 2020 (**A**); Cumulative excess mortality per 1,000,000 inhabitants (**B**). When starting the comparison on 1 August 2020, i.e., when shifting the German excess mortality (dotted blue line) to the UK level of this day, both excess mortalities are nearly identical.

The P-score of the excess mortality calculates the percentage difference between the reported and projected number of deaths during the given weeks. It is displayed in Figure 4A for Germany and the UK. The most striking feature of these curves is the peak of over 100%-meaning twice as many deaths as expected-during the first wave in the UK. For the remaining time period, the two curves move more or less in parallel. In the winter 2020/2021, the P-scores exhibit a wave in both countries, a little earlier in Germany (due to the second wave of the wildtype) than in the UK (due to the Alpha variant). At the end of the year 2021, the Delta variant caused another maximum in the P-score of Germany, while the P-score of the UK normalized towards 0.

Alternatively, one can consider the cumulative excess mortality per million people, shown in Figure 4B. Once again, the shift between the two countries can be assigned to the first wave in March and April 2020. After that, the development of the excess mortality was practically identical in both countries. This becomes obvious if one moves the starting point to 1 August 2020, the day we chose as beginning of the second period. The dotted line in Figure 4B represents the German cumulative excess mortality, assuming equality between the two countries on 1 August 2020, after the first wave. From this time point on, the two curves are very similar and end up at the same point.

**Figure 5:**
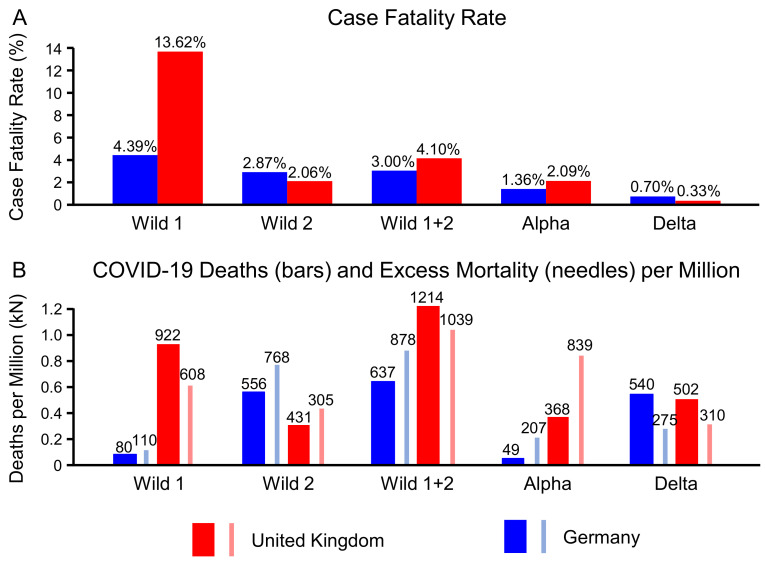
Comparison of Germany and the United Kingdom per COVID-19 wave (Wild 1, Wild 2, Wild 1 + 2, Alpha variant, Delta variant): Case fatality rates (**A**); excess mortality (bars) and number of COVID-19 related deaths (needles) per one million inhabitants (**B**).

The excess mortality includes all causes of deaths. It is therefore of interest to relate the excess mortality during a specific period to the deaths associated with COVID-19. This answers the question how much of the excess mortality is caused by deaths related to SARS-CoV-2. Figure 5B displays both metrics stratified by mutation. The wildtype is estimated to have caused 878 deaths per million in Germany, while the excess mortality was only at 637 deaths per million. In the UK, 1039 deaths per million associated to the wildtype contributed to an excess mortality of 1214. During the Alpha period, the COVID-19 related deaths were more than twice as high as the excess mortality. This relation is reversed by the next mutation. During the Delta period, COVID-19 can only be made responsible for 62% (310 out of 502) of the excess mortality in the UK. In Germany this number dropped to 51% (275 of 540).

Germany has a much higher capacity of ICU beds than the UK. As can be seen in Figure 3A, Germany also used much more ICU beds for COVID-19 patients than the UK at all times, even during the first wave that was much more intense in the UK. As it is not likely that the courses of the disease are generally more severe in Germany, this additional effort should lead to less patients dying from COVID-19.

Assuming identical CFRs one would expect the ratios in Figure 3A,B to be identical. Figure 5A shows that the CFRs varied significantly, hence we get relevant differences between the ratios in Figure 3A,B. During the Delta period, Germany had 4.6 times more COVID-19 patient days on the ICU than the UK. In the same time period, 6.17 people were estimated to have died from COVID-19 per 100 ICU days in Germany, and 13.5 or 2.2 times more in the UK. The ratio of these ratios (4.6/2.2 = 2.1) reproduces the ratio of the CFRs (0.7/0.33 = 2.1), but relating it the ICU patient days gives us another perspective: in the Delta period, Germany invested five times more ICU days into its COVID-19 patients. Despite this huge additional effort and investment, both death-related statistics indicate that it didn’t help: the CFR was twice as high (0.7% versus 0.3%, see Figure 5A), and the excess mortality was higher too (540 versus 502, see Figure 5B).

## 4. Discussion

In the present analysis, we performed a time line analysis of the past four COVID-19 waves in the UK and in Germany and related the results to national prevention measures. The number of patients who died from COVID-19 and the number of ICU beds occupied by COVID-19 patients were used as markers of disease severity. Furthermore, excess mortality in both countries was compared to estimate the net balance of beneficial and disadvantageous measures, including NPI. The number of COVID-19 patients admitted to hospitals could not be used as marker of disease severity because these data are available for the UK but not for Germany.

Our analysis revealed that the COVID-19 epidemiology and death rates in both countries differed markedly in the first wave until July 2020 despite similar prevention measures. The results of various studies have shown that NPI were sufficient in containing the spread of SARS-CoV-2 [24,31,32,33,34], decreasing the reproduction number R, and subsequently preventing COVID-19 related deaths. Therefore, it is surprising that the extent of COVID-19 burden during the first wave was widely different in the UK and in Germany. Although the virus had entered both populations more or less at the same time, and lockdown measures were started concurrently end of March 2020, the consequences for the UK population were much harder than those for the German people. In the first wave the number of reported infections in the UK was one and a half times as big as in Germany, but the number of fatal courses was four and a half times as high. This finding can partially be explained by differences in death rates, raising over-proportionally with increasing infection numbers. More importantly, different test strategies at the beginning of the pandemic strongly influenced these numbers. A recent study [35] estimates the factor between reported infections and actual infections in March 2020 to be 11 in Germany, and 123 in the UK. Taking these factors into account for the first wave, the actual CFR in Germany during the first wave would be 0.4%, compared to 0.11% in the UK. In summary, the difficulties of the UK during the first wave were very likely caused by a huge amount of undetected infections.

In the second wave in winter 2020/2021, in both countries the number of infected individuals was much higher than that of the first wave in spring 2020, suggesting a strong seasonal and meteorological impact on SARS-CoV-2 epidemiology that had been ignored in the early studies when estimating the influence of NPI [24,31,32,33,34]. Surprisingly, in the second wave the scenario between Germany and the UK had reversed. In Germany, the number of infected individuals (2,245,170 vs. 1,422,961), the number of COVID-19 associated deaths (64,471 vs. 29,369), and the CFR (2.87% vs. 2.06%) was higher than the corresponding value in the UK. We hypothesize that the larger number of infected persons during the first wave in the UK resulted in a more extended circulation of the virus within the population between wave 1 and wave 2. Viral circulation resulted in silent infections, inducing (partial) immunity of affected individuals and eventually a diminished strike power of the virus in the second wave. In an earlier study, analyzing the data of 35 European countries, it was shown that this observation can be generalized. The results of that study showed that countries with high incidences and death rates within the first wave had comparably low incidences and death rates within the second wave and vice versa [3]. In agreement with the idea of silent immunization due to unnoticed viral spreading, it was shown that previous infections reduced SARS-CoV-2 reproduction number and incidence in Austria [36].

In both countries, the case fatality rate in the second wave was markedly lower compared to the first wave. This supports the idea of silently generated immunity in-between the first and the second wave. In this “in-between period”, both the number of individuals who died from COVID-19 and the occupation of ICU beds was low. Clearly, in both countries viral spread in the warm period between the first and the second wave only led to few severe courses. Therefore, the immunisation reached during this period comes with very little costs, leading to a long-term positive net balance for the population.

The fact that even increasing incidences in this period, caused by travellers returning to Germany, neither resulted in a higher number of COVID-19 associated deaths nor in an increasing number of patients with COVID-19 on ICUs had been shown earlier [4]. In Germany, the excess mortality during the entire wildtype period was lower than the number of deaths due to COVID-19. This raises the question if the NPI actually prevented dying from COVID-19, or more generally dying from all other causes.

It is worth noting that in both countries the number of deaths peaked at the same time, mid of January 2021, despite the dominance of different genotypes (wild-type virus vs. Alpha variant). This observation confirms that seasonality has a major effect on the epidemiology of the disease.

In conclusion, the data of the first pandemic year suggests that the longer a certain SARS-CoV-2 genotype circulates silently in a population, the less fatal the consequences in the following wave will be. The seasonal situation and the extent of “pre-wave” circulation seem to be key factors for the extent and fatality of the following wave.

These statements are further supported by the course of the third wave caused by the Alpha variant in the UK and in Germany. While the UK was affected in winter 2020/2021, Germany was hit approximately three months later in spring 2021 and the circulation period of the Alpha variant in Germany was more than twice as long as in the U.K. The reason for this difference is due to the fact that the Alpha variant probably evolved in the UK in fall 2020. Obviously, the two factors seasonality and circulation time outweighed the beneficial effect of (partial) immunity against the Alpha variant of individuals that had been in contact with the wildtype virus. In contrast to an earlier study, we found that in the UK COVID-19 burden caused by the Alpha variant was much higher than that caused by the Delta variant. This discrepancy results from methodological differences. In the present analysis, the whole period of Alpha variant activity was examined while in the earlier study the observation period started at the end of March 2021. At that time, the Alpha variant induced third wave was almost over and consequently the major part of Alpha variant caused burden was excluded from that analysis [8].

The delta period dominated from summer 2021 to the end of the year, medical stuff and governments had a year and a half experience dealing with the pandemic, and highly efficient vaccines became widely available. Despite these similar parameters, the two countries acted very differently. In the UK, the lifting of most restrictions on 19 July 2021, led to an extended spread of the virus with approximately 40,000 infections per day. In comparison to this high incidence, the number of deceased patients was relatively low resulting in a CFR of only 0.33%.

In Germany, COVID-19 NPI were kept or even aggravated and prevented infections during the summer months, resulting in comparably low incidences in late summer of 2021 and consequently in low-grade spread in the population. In October 2021, however, when temperatures started to drop, just as the year before, incidences increased exponentially, paralleled by a strong increase of COVID related deaths, resulting in a CFR that was more than twice as high as in the UK. The differences in vaccination rates were very small [37] so that they can’t be used as a plausible explanation.

In the Delta period, the percentage of deceased COVID-19 patients per million inhabitants in Germany was only about 10% smaller than in the UK, but the excess mortality was almost 10% higher. Simultaneously, the number of ICU beds occupied by COVID-19 patients in that period in Germany was about four times as high as in the UK.

This finding indicates that all the efforts made in Germany within the second half of 2021 had no net benefit on the survival of the population. Moreover, it seems plausible to conclude that the German measures, saving the lives of individuals suffering from a vaccine preventable disease, were placed to the debit of patients suffering from diseases not preventable by vaccination. However, this ethical dilemma is systematically ignored in Germany.

As recently concluded, living with COVID-19 is best considered as “optimizing population protection without prohibitive restrictions on their daily lives” [38]. Our analysis shows that the UK came very close to achieving this goal in the second half of 2021. On the other hand, the German approach resulted in more long-lasting restrictions combined with devastating results in November and December 2021.

Therefore, it is reasonable to debate the benefit of NPI. A very early study described that major NPI had a large effect on reducing SARS-CoV-2 transmission [21]. However, others [39] controversially discussed the conclusion made by those authors. On the other hand, there are various later studies demonstrating the efficacy of NPI on infection rates [40,41,42,43,44]. By contrast, in a recent study from Canada it was shown that a high level of NPI had only minor effect on viral containment [45]. Furthermore, a recent study analyzing the effect of NPI on COVID-19 related deaths in 169 countries showed a marginal benefit [46]. Therefore, it seems reasonable that the calculation of the NPI efficacy is non-robust and mainly depends on the general framework and the model used for calculation [47]. A further restriction is that most of the studies currently available estimated the benefit of NPI during the first wave of the wildtype virus. The present results show that NPI in fact appeared to cause short time benefit on the spread of a highly transmissible variant but led to a dreadful scenario with many deaths afterwards. Considering the fact that NPI are accompanied by a multitude of social and economic costs [48,49,50], reasoning for far reaching NPI should be carefully balanced. At least in the warm season, measures as proposed in the Great Barrington Declaration [51] appear more appropriate in Europe than intensified NPI when dealing with a highly transmissible SARS-CoV-2 variant.

Our study has limitations. Since these limitations are primarily due to quality issues of publicly available data, we discuss them in detail.

Our analyses are based on observational data for two European countries. The definition of a COVID-19 related death might vary from country to country, as might the reliability of the reporting itself.

On the other hand, in both countries the case definition of COVID-19 comprises a positive PCR test result. Therefore, a systematic underreporting of COVID-19 in Germany versus the UK and vice versa does not appear very likely [52,53]. Another limitation is the uncertainty about the process used in case of more than one SARS-CoV-2 finding from a single person. A strict procedure might lead to the exclusion of recurrent infections, while accepting all positive test results will cause an overestimation of case numbers. Nevertheless, the providers of the data analyzed here assume that the number of confirmed cases is lower than the number of true infections [54,55].

In addition, it is important to keep in mind that we analyse case fatality rates and not the mortality of the novel coronavirus. Due to the high number of unreported cases, the CFR are an overestimation of mortality.

A further overestimation of the CFR may result from patients dying from other causes like car accidents or heart attacks, coincidentally infected with SARS-CoV-2. In our hospital, we see many positive results in patients who had suffered from COVID-19 weeks or months before the current treatment, and it seems reasonable to assume that those patients die with and not from a SARS-CoV-2 infection. On the other hand, finding of SARS-CoV-2 RNA is only possible if a contact to the virus had happened. Various preexisting diseases increase the probability for fatal courses, e.g., heart diseases [56,57,58,59]. Often it is difficult to discriminate if SARS-CoV-2 infection contributed to the death of a patient, e.g., if a still SARS-CoV-2 positive patient with a previous infection dies due to a heart attack. In such a setting, it is impossible to exclude that a virus induced activation of coagulation contributed to the formation of a thrombus.

In both countries, physicians are committed to notify the death from COVID-19. In Germany, only the death or the assumption that a death had been caused by COVID-19 must be notified [60]. The comparison with the UK is complicated by the fact that the UK health authorities changed the reporting requirements in April 2020. In the UK, a notification is required if COVID-19 was documented as a direct or underlying cause of death, even in the absence of a positive COVID-19 test [61]. This difference might contribute to higher death rates in the UK compared to Germany. The impact of legal regulations on COVID-19 death statistics was demonstrated in detail earlier [62].

The analysis of excess mortality is therefore an important additional aspect. However, calculating the excess mortality is not a completely standardized procedure and the results depend not only on the raw data but also on the algorithm used. Apart from the World Mortality Dataset used in the present analysis a multitude of algorithms are available [63]. In a recent preprint [64], six algorithms were used to calculate the excess mortality of 33 high-income countries and big differences were obtained depending on the algorithm. Another finding of that study was that age adjustment is a precondition to achieve good data quality, as reported earlier [63]. The World Mortality Dataset imports data from two age disaggregating sources fulfilling this requirement. In addition, backward revisions are used for the World Mortality Dataset, improving the quality of preliminary data. In summary, the excess mortality data presented herein rely on sophisticated and transparent procedures, but one needs to be aware of its limitations.

Apart from the number of deaths and the excess mortality, the number of COVID-19 patients treated on an ICU was examined as marker of disease severity. It has to be mentioned that not every SARS-CoV-2 positive patient on an ICU was treated because of severe COVID-19. Frequently SARS-CoV-2 was the leftover of a previous infection and therefore detected incidentally. As in the UK fewer COVID-19 patients had been treated on ICUs with a higher rate of deceased patients, it seems plausible that in Germany much more infections were found accidentally.

Finally, the testing strategies vary a lot from country to country, and changed within each country over the course of the pandemic. This introduces a source of variation to the incidence data that we cannot control for.

## 5. Conclusions

In summary, the comparison of the four COVID-19 waves between the UK and Germany allows the following conclusions:

- Silent spread of SARS-CoV-2 induces immunity in affected individuals lowering extension and fatality of the following wave. The more individuals are silently affected the more pronounced the benefit.
- There is a strong seasonal association of SARS-CoV-2 epidemiology and fatality. The seasonal effect can outweigh other factors and lead to reduced efficiency of NPI.
- In the UK and in Germany, NPI in the warm season were counterproductive to achieve mild courses within the following wave.

More research is needed to investigate the right balance between infection-controlling restrictions and reaching population immunity in a controlled manner.

## Figures and Tables

**Table 1:** Comparison of Germany and the UK regarding some general population parameters, hospital beds and ICU beds, and costs of the public health system (all data for 2020). The general metrics are similar in both countries. However, Germany has almost three times more hospital beds per person and five times more ICU beds per person than the UK.

	Germany	UK
	Population Parameters	
Population (million)	83.1	68.2
Median Age (years)	46.6	40.8
Percentage over 65 years	21.5%	18.5%
Mean Life Expectancy (years)	81.3	81.3
	Public Health Costs	
Total (billion):	€411	€320 (269 Pound)
Costs per person:	€5298	€4735 (3944 Pound)
Percentage of GDP:	13.1%	12.8%
	Hospital beds	
Total:	486,700	141,000
Beds per 1000 people:	5.8	2.1
	ICU beds	
Total:	25,000	4500
ICU Beds per 100,000 people:	29.8	6.6

**Table 2:** Summary statistics of COVID-19 related metrics in Germany and the UK, stratified by SARS-CoV-2 variant. A period ended when more than 50% of sequenced viruses were attributed to the new variant. To separate the first two waves caused by the wildtype of the virus, 1 August 2020, was chosen.

Germany								
Mutation/Wave	Period	Deaths	Cases	CFR	Deaths per Million	ICU sum	ICU per 1000 cases	Deaths per 100 ICU days
Wild 1	1 March 2020–31 July 2020	9230	210,320	4.39%	110	151,841	722.0	6.1
Wild 2	1 August 2020–1 March 2021	64,471	2,245,170	2.87%	768	562,090	250.4	11.5
Wild 1 + 2	1 March 2020–1 March 2021	73,701	2,455,490	3.00%	878	713,931	290.7	10.3
Alpha	2 March 2021–21 June 2021	17,367	1,275,050	1.36%	207	367,562	288.3	4.7
Delta	22 June 2021–27 December 2021	23,059	3,297,779	0.70%	275	373,685	113.3	6.2
**UK**								
Mutation/Wave	Period	Deaths	Cases	CFR	Deaths per Million	ICU sum	ICU per 1000 cases	Deaths per 100 ICU days
Wild 1	1 March 2020–31 July 2020	41,491	304,732	13.62%	608	146,616	481.1	28.3
Wild 2	1 August 2020–6 December 2020	29,369	1,422,961	2.06%	431	85,719	60.2	34.3
Wild 1 + 2	1 March 2020–6 December 2020	70,860	1,727,693	4.10%	1039	232,335	134.5	30.5
Alpha	7 December 2020–16 May 2021	57243	2,738,835	2.09%	839	256,737	93.7	22.3
Delta	17 May 2021–11 December 2021	21,128	6,362,960	0.33%	310	156,161	24.5	13.5

## Data Availability

The data used in this study is publicly available from the website “Our World in Data” (www.ourworldindata.com/coronavirus accessed on 15 March 2022).
